# Experience of the Time to Change programme in England as predictor of mental health service users' stigma coping strategies

**DOI:** 10.1017/S204579601600041X

**Published:** 2016-07-28

**Authors:** G. Sampogna, I. Bakolis, E. Robinson, E. Corker, V. Pinfold, G. Thornicroft, C. Henderson

**Affiliations:** 1 Department of Psychiatry, University of Naples SUN, Naples, Italy; 2 Health Services and Population Research Department, Institute of Psychiatry, Psychology and Neuroscience, King's College London, London, UK; 3 Department of Biostatistics, Institute of Psychiatry, Psychology and Neuroscience, King's College London, London, UK; 4 Unit for Social and Community Psychiatry, WHO Collaborative Centre for Mental Health, London E13 8SP H, UK; 5 McPin Foundation, 32-36 Loman Street, London SE1 0E, UK

**Keywords:** Coping strategy, discrimination, mental health, public health, stigma

## Abstract

**Aims.:**

In the field of stigma research, an area of interest is the coping strategies that mental health service users can use in response to discriminatory experiences. As a part of the evaluation of the Time to Change (TTC) anti-stigma programme, the Viewpoint telephone survey was run annually in order to assess service users' reported levels of discrimination and selected coping strategies. The study aim is to test the extent to which experience of TTC programme is a positive predictor of selected coping strategies.

**Methods.:**

Telephone interview surveys carried out by peer interviewers were conducted annually. ‘Educating others’ and ‘challenging’ coping strategies were assessed alongside anticipated and experienced discrimination.

**Results.:**

During 2011–2014, 3903 mental health service users were interviewed. Participants more often adopted the ‘educating others’ strategy (2.31 ± 0.01) than the ‘challenging’ strategy (2.15 ± 0.02) (*p* < 0.001). On the other hand, those who participated in campaign activities endorsed ‘challenging’ more frequently than people who were not aware of TTC (2.78 ± 1.23 *v.* 2.09 ± 1.08, *p* < 0.001). According to the multi-variate linear regression model, we found that being actively involved in TTC activities (OR = 0.74, CI: 0.29–1.19; *p* < 0.05), having a diagnosis of a depressive disorder (OR = 0.20, CI: 0.04–0.36; *p* < 0.05) or personality disorder (OR = 0.23, CI: 0.04–0.43; *p* < 0.05) were good predictors of endorsing a ‘challenging’ strategy even after adjusted for confounding variables.

**Conclusions.:**

A positive relationship between participating in the TTC programme and using the ‘challenging’ strategy was found. There is still a need to disentangle the complex association between these two coping strategies and the role of anti-stigma campaigns, promoting further local activities led by service users and carers' as well as all others stakeholders' associations.

## Introduction

It has been well documented that people with mental health problems report high levels of discrimination (Sibitz *et al.* 2011; Evans-Lacko *et al.* 2012; Farrelly *et al.* 2014; Griffiths *et al.* 2014). There is an increasing effort to try to end discrimination towards people affected by mental health problems (Heinz *et al.* 2015; Mehta *et al.* 2015; Thornicroft *et al.* 2015; Wahlbeck, 2015; Weissman, 2015), with the promotion of anti-stigma programmes worldwide such as ‘Like Minds, Like Mine’ (Vaughan & Hansen, 2004; Thornicroft *et al.* 2014), ‘Opening Minds’ (Sartorius, 2014; Stuart *et al.* 2014), and ‘Open the Doors’ (Rüsch *et al.* 2005; Gaebel *et al*. 2008; NHS Scotland, 2008; Thornicroft *et al.* 2008; Evans-Lacko *et al.* 2012; Evans-Lacko *et al.* 2014*a*, *b*).

The Time to Change (TTC) anti-stigma programme, England's largest programme aimed at reducing stigma and discrimination against people with mental health problems, started in 2007 (http://www.time-to-change.org.uk/) (Henderson *et al.* 2013). One of the main strategies of the TTC programme was to encourage service user leadership including events with the active involvement of mental health service users (i.e. ‘Get moving’, ‘Living libraries’ and ‘Education not discrimination’ activities) (Evans-Lacko *et al.* 2013; Friedrich *et al.* 2013). As part of the evaluation of TTC, and specifically to assess the effect of the programme on people with mental health problems, the Viewpoint survey was run annually to assess individuals' experience of discrimination within the previous 12 months (Henderson & Thornicroft, 2009; Henderson *et al.* 2012; Corker *et al.* 2013; Evans-Lacko, 2014*a*). Moreover, in the field of overcoming discrimination, it is important to assess service users' responses to discriminatory experiences, which constitute the process of coping (Mendoza-Denton *et al.* 2006; Hinshaw, 2007). Coping strategies may represent a way for service users to reduce the level of discrimination they experience (Farrelly *et al*. 2015).

According to Stuart *et al.* (2012), the most important contribution to the conceptualization and measurement of stigma coping is the work by Link *et al.* (2001). Link distinguished several coping orientations: ‘secrecy’, described as concealing labelling information; ‘education’, consisting in providing information to counter stereotypes; ‘withdrawal’, defined as avoiding potential rejecting situations; ‘distancing’, cognitive separation of the potentially stigmatised person from the stigmatised group; and ‘challenging’, active confrontation of stigmatising behaviour (Link *et al.* 2014). The TTC programme aims to enhance the ‘challenging’ and ‘educating’ strategies in people with mental health problems. Since 2011, to provide a baseline for the second phase of the TTC programme – which began in October 2011 – questions on coping with stigma based on Link *et al*. conceptualization (1991, 2002) were added.

Coping strategies are far from being uniform among mental health service users. Research has found their endorsement is influenced by socio-demographic characteristics, variables related to mental health problems, and by the person's social functioning (Major & Townsend, 2010; Moses, 2015).

To the best of our knowledge, no previous studies have evaluated whether anti-stigma programmes are possible predictors of coping strategies adopted by people with mental health problems.

Our primary hypothesis was that having actively participated in programme activities would be more strongly associated with the use of both ‘challenging’ and ‘educating’ coping strategies, than just having seeing some of the social marketing campaign, as the programme seeks to both educate and challenge.

## Methods

From 2008 to 2014, telephone interview surveys (the Viewpoint survey) were conducted annually. Each year, five National Health Service (NHS) mental health trusts across England were selected to take part. Different trusts and/or different regions within the same trusts were selected each year. Participants were recruited through NHS mental health trusts (service provider organisations). The target sample was 1000 individual interviews in each year. The inclusion criteria were: age between 18 and 65 years; having a diagnosis of any mental disorder; being in contact with the specialist mental health services in the previous 6 months. Patients who were not currently living in the community (e.g., were in prison or hospital) were excluded as well as patients with a diagnosis of dementia. A specific part of the Viewpoint survey was the use of interviewers with lived experience of mental health problems (Hamilton *et al.* 2011).Telephone interviewers were trained and supervised by members of the research team. Allocation of participants to interviewers was based on interviewer availability. The detailed methodology is reported elsewhere (Hamilton *et al.* 2011; Corker *et al.* 2013; Henderson *et al.* 2014).

### Assessment tools

The primary outcome of the study was to assess the possible predictive role of participation in the TTC programme on mental health users' reported coping strategies as per Link *et al*. (2002). Coping strategies were assessed through a modified version of the scale developed by Link *et al.* (2002). According to the main characteristics of the TTC programme, based on the active involvement and participation of people with mental health problems, the ‘challenging’ and ‘educating’ coping strategies were evaluated (Henderson *et al.* 2013). The ‘challenging’ strategy is defined as people's orientation to confronting prejudice and discrimination. It assesses how likely respondents are to challenge stigmatising behaviour when it occurs or to disagree with people who make stigmatising statements. Items are scored on a 5-level Likert scale, ranging from 1 = never to 5 = often. The global score of the scale is the mean of five items, with higher score indicating higher endorsement of the strategy. The ‘educating’ strategy is described as the respondents' orientation to educating others in order to reduce the possibility of rejection. This subscale comprises three items, each item ranges on a 4-level Likert score, from 1 = strongly agree to 4 = strongly disagree. The global score of the scale is the mean of three items, with higher scores indicating higher level of experience of using this strategy. As suggested by Link *et al.* (2002), the 2.5 midpoint of each subscale was considered as cut-off for evaluating whether the coping strategy was used or not.

Regarding the evaluation of the experience of the TTC programme, participants were asked a question, which had three possible answers: have seen some publicity; have participated in some activities; or have not seen any publicity of TTC programme. This variable was managed as a categorical variable, considering ‘have not seen any publicity of TTC programme’ as the reference category.

The Discrimination and Stigma Scale (DISC-12) was used to measure both experienced and anticipated discrimination (Brohan *et al.* 2013). The DISC was administered by the interviewer by telephone. It consists of 22 items on negative, mental health-related experiences of discrimination (covering 21 specific life areas, plus one for ‘other’ experience) and four items concerning anticipated discrimination. Each item is scored on a 4-point scale from ‘not at all’ to ‘a lot’.

The Resource Generator-UK (RG-UK) was used to evaluate the participant's access to social resources, a method of measuring social capital (Webber & Huxley, 2007). Social capital is defined as available resources accessible to the person through trusting and reciprocal relationships within the social networks (Henderson *et al.* 2014). The RG-UK evaluates the capacity of participants to obtain access to several skills and resources within their social network within 1 week, if they needed it. The items are grouped into 4 subscales, each representing a domain of social capital to which an individual may have access: domestic resources, personal skills, expert advice and problem-solving resources. A higher score indicates higher possibility to access to social capital.

In the telephone survey, participants' main socio-demographic and clinical data (i.e., age, gender, ethnicity, clinical diagnosis and employment status) were collected. These items were included in the analysis due to having potential impact on participant's coping strategy.

### Statistical analysis

Descriptive statistics were performed in order to describe the overall sample and non-parametric tests were employed to test the association between coping strategies and experience with the TTC programme. In order to test the association between the experience with TTC programme on the ‘educating’ and ‘challenging’ coping strategies, linear regression models were employed and adjusted for: age, gender, psychiatric diagnosis, working status, ethnicity, RG-UK score, anticipated and experienced discrimination. In order to adjust for possible regional effects, NHS mental health trusts were grouped into four categories (North of England; England Midlands and East; South of England; and London) (http://www.healthcheck.nhs.uk/interactive_map) and also included in the models.

All confounders were identified *a priori* by previous studies (Miller & Kaiser, 2001; Corrigan & Watson, 2002; Mendoza-Denton *et al.* 2006; Ilic *et al.* 2012; Evans-Lacko *et al.* 2014*a*, *b*).

An interaction term was also added to the regression models between the time-point (each year of the Viewpoint campaign) and the exposure to the TTC programme variable, in order to identify any effect modification of the association between ‘Challenging’ and ‘Educating’ strategies.

## Results

### Descriptive results

During the period 2011–2014, 3903 mental health service users were interviewed. Respondents were disproportionately female (62.2%, *n* = 2430), with a mean (s.d.) age of 44.3 (11.4) years, and unemployment was reported by almost half of the sample (49.3%; *N* = 1922). The most commonly reported diagnoses were mood disorders (depression, 29.8%, *n* = 1059; bipolar disorder, 20.7%, *n* = 734), followed by schizophrenia/schizoaffective disorders (17.2%, *n* = 610); anxiety disorders (12.1%, *n* = 431); personality disorders (9.3%, *n* = 329); and other mental disorders (11.0%, *n* = 389) (see Table 1).

**Table 1.** Socio-demographic characteristics of the sample of mental health users interviewed during the period 2011–2014

	Global sample	Year	Year	Year	Year
	*N* = 3903	2011	2012	2013	2014
		*N* = 1014	*N* = 1003	*N* = 984	*N* = 902
Experience with the TTC programme, % (*n*)*					
Have not seen any publicity	72.7 (2823)	81.0 (820)	72.4 (723)	69.4 (678)	67.1 (602)
Seen some publicities	24.5 (953)	16.8 (170)	26.2 (262)	26.6 (260)	29.1 (261)
Participated in some activities	2.8 (110)	2.3 (23)	1.4 (14)	4.0 (39)	3.8 (34)
Region, % (*n*)					
North	40.2 (1562)	41.9 (426)	53.0 (532)	45.2 (445)	19.0 (171)
Midlands	19.0 (740)	38.8 (394)	0	11.1 (109)	26.6 (240)
South	24.6 (957)	0	31.2 (313)	27.8 (274)	41.6 (375)
London	16.1 (627)	19.3 (196)	15.8 (159)	15.9 (157)	12.9 (116)
Age, mean (s.d.)	44.3 (11.4)	45.0 (11.2)	43.7 (11.2)	44.5 (11.3)	44.0 (11.9)
Gender, % (*n*)					
Male	37.6 (1462)	40.5 (411)	38.6 (387)	37.1 (365)	33.9 (306)
Female	62.2 (2416)	59.3 (602)	61.5 (617)	62.5 (616)	66.0 (595)
Transgender	0.2 (7)	0.2 (2)	0	0.4 (4)	0.1 (1)
Ethnicity, white, % (*n*)	90.9 (3503)	89.6 (904)	90.9 (898)	90.1 (886)	93.2 (833)
Employment status, % (*n*)					
Employed	23.9 (927)	23.6 (239)	22.2 (222)	21.0 (207)	29.4 (265)
Studying/training/volunteering/other	18.9 (735)	19.3 (196)	23.8 (238)	12.3 (121)	20.4 (184)
Unemployed	49.3 (1914)	47.8 (485)	47.7 (478)	59.1 (582)	41.9 (377)
Retired	7.9 (305)	9.4 (95)	6.4 (64)	7.6 (75)	8.2 (74)
Diagnosis, % (*n*)					
Anxiety disorders	12.1 (429)	9.1 (82)	9.4 (86)	14.1 (130)	16.5 (133)
Bipolar disorders	20.7 (731)	20.3 (184)	23.8 (218)	19.6 (181)	18.7 (151)
Depression	29.8 (1051)	34.4 (311)	28.0 (257)	28.7 (625)	28.0 (226)
Personality disorders	9.3 (328)	6.1 (55)	7.7 (71)	10.6 (98)	13.0 (105)
Schizophrenia and schizoaffective disorders	17.2 (607)	15.7 (142)	21.8 (200)	17.1 (158)	13.7 (110)
Other	11.0 (387)	14.5 (131)	9.3 (85)	10.0 (92)	10.1 (81)
Resource Generator (RG), total score, mean (s.d.)	13.3 (6.0)	13.3 (6.1)	13.3 (6.0)	12.6 (5.9)	14.0 (6.1)
RG domestic resources, mean (s.d.)	3.9 (2.0)	4.0 (2.0)	3.8 (2.0)	3.7 (2.0)	4.0 (2.0)
RG expert advice, mean (s.d.)	4.0 (2.4)	4.2 (2.4)	4.1 (2.4)	3.8 (2.3)	4.1 (2.4)
RG personal skills, mean (s.d.)	2.7 (1.6)	2.8 (1.6)	2.6 (1.6)	2.5 (1.6)	2.9 (1.7)
RG problem solving skills, mean (s.d.)	2.8 (1.3)	2.9 (1.3)	2.8 (1.3)	2.7 (1.2)	2.9 (1.3)
Anticipated discrimination, mean (s.d.)	58.3 (33.4)	58.5 (33.9)	62.2 (33.6)	57.1 (33.1)	54.6 (32.7)
Experienced discrimination, mean score (s.d.)	31.8 (22.8)	30.9 (23.1)	34.2 (23.6)	32.4 (22.0)	29.7 (22.1)
Challenging coping strategy, mean score (s.d.)	2.2 (0.02)	2.2 (1.7)	2.1 (1.0)	2.1 (1.1)	2.1 (1.1)
Educating coping strategy, mean score (s.d.)	2.3 (0.01)	2.1 (0.7)	2.9 (0.6)	2.1 (0.6)	2.1 (0.6)

* Missing data, *N* = 17.

We found a slightly higher reported use of the educating strategy (40.9%, *n* = 1596) compared with the challenging strategy (38.7%, *n* = 1513) (*p* < 0.001).

As regards service users' awareness or involvement in TTC, the proportion of people who actively participated in the campaign ranged from 2.3% in 2011 to 3.8% in 2014. However, there was an increase in the proportion stating they had seen at least some publicity about TTC, from 17% in 2011 to 29.1% in 2014.

As regards the challenging strategy, respondents participating in programme activities 2011–2014 reported using it more frequently than people who had never heard about TTC (57.3% *v.* 37.0%; *p* < 0.001) as well as compared with people who had only seen some publicity (57.3% *v.* 41.2%; *p* < 0.001) (Table 2).

**Table 2.** Coping strategies and level of experience of the TTC campaign

	No	Seen some publicity	Participating in some activities	*p*
Educating coping strategy, mean (s.d.)	2.34 (0.70)^a^	2.29 (0.73)^b^	1.74 (0.79)^a^^,^^b^	<0.001
Challenging coping strategy, mean (s.d.)	2.09 (1.08)^a^^,^^c^	2.24 (1.08)^b^^,^^c^	2.78 (1.23)^a^^,^^b^	<0.001

One-way ANOVA, with Bonferroni corrections.

a Having never heard about TTC *v.* Having participating in TTC, *p* < 0.001.

b Having seen some publicity *v.* Having participating in TTC, *p* < 0.001.

c Having never heard about TTC *v.* Having seen some publicity of TTC, *p* < 0.001.

For the educating coping strategy, mental health users having seen some publicity of TTC reported a higher endorsement of the strategy compared with those actively involved in the campaign (2.29 ± 0.73 *v.* 1.74 ± 0.79, *p* < 0.001). Respondents not aware of TTC also endorsed this strategy more frequently compared with people participating in some programme activities (2.34 ± 0.70 *v.* 1.74 ± 0.79, *p* < 0.001) (Table 2).

### Association between TTC and coping strategies

We found that having actively participated in the campaign activities represented a good predictor of endorsing the challenging coping strategy (OR = 0.74, CI: 0.29–1.19; *p* < 0.05), adjusted for the impact of the year of the campaign, the geographical region and for the other socio-demographic confounding variables. Moreover, just having seen some publicity had a slight positive impact on endorsement of the challenging strategy, but it did not reach the level of statistical significance (see Table 3). Interestingly, we found that each psychiatric diagnosis had a different impact on the endorsement of the challenging coping strategy. In particular, people with depressive (OR = 0.20, CI: 0.04–0.36; *p* < 0.05) or personality disorders (OR = 0.23, CI: 0.04–0.43; *p* < 0.05) had a slightly higher probability of adopting such a strategy. Moreover, female participants (OR = 0.09, CI: 0.00–0.19; *p* < 0.05) and people with higher resources at the RG-UK scale (OR = 0.02, CI: 0.02–0.03; *p* < 0.05) were more prone to adopt the challenging strategy. Results of the multivariate multiple regression models are shown in Table 3.

**Table 3.** Multivariate multiple regression models – weighted sample

	Educating subscale	Challenging subscale
	Coefficient (95% CI)	Coefficient (95% CI)
Impact of TTC		
(reference) Have not seen any publicity	–	–
Seen some publicity	−0.09 (−0.19 to 0.01)	0.09 (−0.08 to 0.27)
Participated in some activities	−0.72 (−0.88 to −0.55)*	0.74 (0.29 to 1.19)*
Year of the campaign		
2011	−0.01 (−0.10 to 0.08)	0.04 (−0.12 to 0.20)
2012	0.68 (0.59 to 0.78)*	−0.01 (−0.19 to 0.16)
2013	−0.05 (−0.13 to 0.03)	0.08 (−0.09 to 0.25)
(reference) 2014	–	–
Impact of TTC* year of the campaign		
Seen some publicity*2011	−0.06 (−0.21 to 0.08)	−0.07 (−0.37 to 0.22)
Seen some publicity*2012	0.27 (0.12 to 0.42)*	−0.02 (−0.27 to 0.24)
Seen some publicity*2013	0.04 (−0.09 to 0.17)	−0.35 (−0.63 to −0.08)*
Participated in some activities*2011	0.19 (−0.22 to 0.61)	−0.22 (−1.05 to 0.60)
Participated in some activities*2012	1.29 (0.93 to 1.66)*	−0.46 (−1.08 to 0.16)
Participated in some activities*2013	0.15 (−0.08 to 0.38)	−0.26 (−0.91 to 0.38)
Clinical diagnosis, ref. Others	–	–
Anxiety disorders	0.05 (−0.07 to 0.16)	0.19 (−0.02 to 0.39)
Bipolar disorder	0.01 (−0.08 to 0.10)	0.15 (−0.006 to 0.31)
Depression	0.03 (−0.06 to 0.11)	0.20 (0.04 to 0.36)*
Personality disorders	−0.07 (−0.18 to 0.04)	0.23 (0.04 to 0.43)*
Schizophrenia and schizoaffective	0.11 (0.01 to 0.22)*	−0.001 (−0.18 to 0.18)
RG total score	−0.01 (−0.02 to −0.01)*	0.02 (0.02 to 0.03)*
DISC Total score	−0.002 (−0.003 to −0.001)*	0.01 (0.01 to 0.02)*
Anticipated discrimination	0.002 (0.001 to 0.002)*	0.0003 (−0.001 to 0.001)

*Significance level set at *p* < 0.005, all models adjusted for region, gender age, working status, ethnicity.

Wald test, educating: impact of TTC* year of the campaign, *p* < 0.001.

Wald test, challenging: impact of TTC* year of the campaign, *p* < 0.005.

As regards the educating coping strategy, mental health users actively participating in the programme's activities scored lower on the educating coping subscale (OR = −0.72, CI:−0.88 to −0.55; *p* < 0.05), also adjusting for confounding variables as the year of the Viewpoint survey. The clinical diagnosis seemed to have an impact on the endorsement of educating coping strategy, and people with a diagnosis of schizophrenia/schizoaffective disorders showed a higher probability of adopting such a strategy (OR = 0.1, CI: 0.01–0.22; *p* < 0.05). Interestingly, people reporting higher levels of anticipated discrimination were more likely to educate others on mental health topics (OR = 0.002, CI: 0.001–0.002; *p* < 0.05) (Table 3).

## Discussion

Although there are several anti-stigma programmes ongoing in different countries, these have not to date explored the possible relationship between level of participation in such programmes and endorsement of coping strategies by service users. This study represents the first effort in Europe to test the possible role of an anti-stigma campaign as one of the potential predictors of mental health service users' ‘challenging’ and ‘educating’ coping strategies.

The hypothesis of the positive association between active involvements in TTC activities on two selected coping strategies was partially confirmed by our results. In particular, mental health service users actively participating in the anti-stigma project report that, they challenge stigma more than those not aware of TTC. Such a finding is consistent with the characteristics of TTC, which features contact-based activities between mental health users and members of the general population (Henderson & Thornicroft, 2009; Henderson *et al.* 2013). On the other hand, longitudinal studies are needed in order to evaluate the long-term endorsement of the challenging strategy, beyond the duration of the anti-stigma campaign activity, which represents the ideal environment to apply it (Sartorius, 2010).

Moreover, possible explanatory variables having an impact on challenging coping strategy were identified, as the role of female gender and of social capital. In particular, as regards gender, our data are in line with the results of the ‘Like Minds, Like Mine’ programme, which found that women were more likely than men to say they had coped with stigma and discrimination (Ministry of Health, New Zealand, 2011). Moreover, we found that social capital has a positive impact on the endorsement of the challenging coping strategy. This positive impact of social capital is in line with recent studies in the field of stigma showing that social capital is related with empowerment, impacting also on self-stigma (Brohan *et al*. 2010, 2011; Lanfredi *et al*. 2015; Corker *et al*. 2016).

On the other hand, our results did not confirm the hypothesis that participating in the programme activities is associated with more use of the educating coping strategy. To date, the total sample of mental health users interviewed reported adopting the educating strategy more frequently than the challenging one. This general trend in the sample may have reduced the possibility to evaluate the potential predictive role of the participation in the TTC programme on the adoption of such strategy. This finding deserves further study, considering that contact-based education strategies have been found to be more effective than protest for overcoming stigma in the adult population, while for adolescents education strategy seems more effective (Corrigan *et al.* 2012).

Moreover, we searched for the possible explanatory role of discrimination on coping strategies. It seems that the relationship between coping strategies and anticipated discrimination is very complex, considering that Brain *et al.* (2014) found that anticipated discrimination was inversely associated with coping strategies. Such association was not confirmed in our study. A preliminary explanation could be due to the fact that some mental health users reporting anticipated discrimination want to take an action to prevent it, and so discrimination itself represents a good predictor for endorsing such coping strategies. Of course, this suggestion is tentative and future confirmatory studies are needed in order to disentangle and deepen the knowledge on the causal relationship between discrimination and coping responses.

Another interesting result is related to the increasing rate of mental health users stating they had ‘seen some publicity from the TTC programme’. It seems that since the start of the programme, mental health services' users are becoming more aware of TTC, findings in line with figures based on the social marketing campaign of the TTC (Evans-Lacko *et al.* 2013).

The educating and challenging strategies were chosen as focus of the present study, as these strategies are related to the aims of the TTC programme (Henderson *et al*. 2013). In particular, the main feature of the TTC campaign activities was to invite people with mental disorders to participate in various sporting activity (i.e., ‘Get Moving!’) or in discussions on their diagnosis (e.g., ‘Living Libraries’) (London & Evans-Lacko, 2010). Taking this into account, the study focussed on the possible impact of the TTC activities on these two specific coping strategies. This methodological choice also avoided further increasing the length of the interview and the participants' burden in taking part.

In the present study, a cross-sectional design was adopted. This methodological choice could have limited our findings, considering that coping strategies are the results of a dynamic process (Hinshaw, 2007). Moreover, we were not able to detect to which extent the coping strategies are directly associated with exposure to the programme. This is due to the fact that the relevant questions were added in 2011, while the survey was launched in 2008. It could be that service users – already used to challenging stigma and educating others – were more likely to get involved in TTC. We addressed this limitation adjusting our regression analysis according to the years of the campaign and to the relevant interaction term.

Finally, a selection bias in respondents could have limited the generalisability of the present findings. It could be that those experiencing higher levels of discrimination were more likely to take part of the survey and were thus overrepresented. In fact, in our sample we found that people reporting higher levels of anticipated and experienced discrimination were more prone to adopt the challenging strategies. This could in part be due to them having more opportunities to do so.

The TTC anti-stigma programme represents one of the major efforts in England to end mental health stigma and discrimination. Evidence is encouraging on the positive relationship between participating in the activities of the TTC programme and using of challenging coping strategy. Such results shed light on the possible way forward in the field of fighting and overcoming stigma, although some gaps need to be filled in. To improve the research evidence base, a higher number of service users will need to be involved in the campaign activities, and the evaluation should have a longitudinal design. A possible solution could be represented by the promotion of further local activities led by service users and carers' as well as all others stakeholders' associations and by the more active involvement of mental health services (Fiorillo *et al.* 2013; Copeland *et al*. 2014; Forsman *et al.* 2015; Wykes *et al.* 2015).

## Availability of Data and Materials

The DISC measure is available subject to terms and conditions at http://www.kcl.ac.uk/ioppn/depts/hspr/research/ciemh/cmh/CMH-Measures.aspx. The Viewpoint dataset is not available. Data sharing would first require an application to the UK's Health Research Authority, which the research team does not have the capacity to do now that the Viewpoint survey has been discontinued.
